# Evaluating Perinatal Health in Europe: A Comparison of Routine Population Birth Data Sources

**DOI:** 10.1111/ppe.13178

**Published:** 2025-03-11

**Authors:** Marianne Philibert, Mika Gissler, Oscar Zurriaga, Serena Donati, Zeljka Drausnik, Günther Heller, Alison Macfarlane, Ashna Mohangoo, Luule Sakkeus, Vlad Tica, Petr Velebil, Jeannette Klimont, Lisa Broeders, Tonia A. Rihs, Jennifer Zeitlin, the Euro‐Peristat Research Group, Alex Farr, Sophie Alexander, Wei‐Hong Zhang, Rumyana Kolarova, Željka Draušnik, Theopisti Kyprianou, Vasos Scoutellas, Petr Velebil, Laust Hvas Mortensen, Luule Sakkeus, Liili Abuladze, Mika Gissler, Béatrice Blondel, Anne Chantry, Catherine Deneux‐Tharaux, Mélanie Durox, Alice Hocquette, Marianne Philibert, Jennifer Zeitlin, Guenther Heller, Aris Antsaklis, István Sziller, Johanna Gunnarsdóttir, Karen Kearns, Marina Cuttini, Janis Misins, Irisa Zile, Jelena Isakova, Aline Lecomte, Audrey Billy, Jessica Pastore, Miriam Gatt, Jan Nijhuis, Kari Klungsoyr, Katarzyna Szamotulska, Ewa Mierzejewska, Henrique Barros, Mihai Horga, Jan Cap, Miha Lučovnik, Ivan Verdenik, Oscar Zurriaga, Karin Källén, Anastasia Nyman, Tonia Rihs, Alison Macfarlane, Sonya Scott, Kirsten Monteath, Siobhán Morgan, Joanne Murphy

**Affiliations:** ^1^ Obstetrical Perinatal and Pediatric Epidemiology Université Paris Cité, Inserm Paris France; ^2^ THL Finnish Institute for Health and Welfare Helsinki Finland; ^3^ Karolinska Institute Stockholm Sweden; ^4^ Preventive Medicine and Public Health Department University of Valencia Valencia Spain; ^5^ FISABIO, Rare Diseases Research Area and Mixed Unit FISABIO‐UVEG Valencia Spain; ^6^ National Center for Disease Prevention and Health Promotion—Istituto Superiore di Sanità Italian National Health Institute Rome Italy; ^7^ Division of Public Health, Croatian Institute of Public Health Zagreb Croatia; ^8^ Institute for Quality Assurance and Transparency in Healthcare IQTIG Berlin Germany; ^9^ Maternal and Child Health and Research Centre, City University of London London UK; ^10^ Foundation for Perinatal Interventions and Research in Suriname (PeriSur) Paramaribo Suriname; ^11^ Estonian Institute for Population Studies Tallinn University Tallinn Estonia; ^12^ East European Institute for Reproductive Health, Faculty of Medicine University “Ovidius” Constanţa, Romanian Academy of Scientists Constanta Romania; ^13^ Institute for the Care of Mother and Child Prague and 3rd Medical School of Charles University Prague Czech Republic; ^14^ Statistics Austria Vienna Austria; ^15^ Perined Utrecht the Netherlands; ^16^ Federal Statistical Office Neuchâtel Switzerland

**Keywords:** birth data, Europe, international comparisons, perinatal indicators, pregnancy

## Abstract

**Background:**

International comparisons of population birth data provide essential benchmarks for evaluating perinatal health policies.

**Objectives:**

This study aimed to describe routine national data sources in Europe by their ability to provide core perinatal health indicators.

**Methods:**

The Euro‐Peristat Network collected routine national data on a recommended set of core indicators from 2015 to 2021 using a federated protocol based on a common data model with 16 data items. Data providers completed an online questionnaire to describe the sources used in each country. We classified countries by the number of data items they provided (all 16, 15–14, < 14).

**Results:**

A total of 29 out of the 31 countries that provided data responded to the survey. Routine data sources included birth certificates (15 countries), electronic medical records (EMR) from delivery hospitalisations (16 countries), direct entry by health providers (9 countries), EMR from other care providers (7 countries) and Hospital Discharge Summaries (7 countries). Completeness of population coverage was at least 98%, with 17 countries reporting 100%. These databases most often included mothers giving birth in the national territory, regardless of nationality or place of residence (24 countries), whereas others register births to residents only. In 20 countries, routine sources were linked, including linkage between birth and death certificates (16 countries). Countries providing all 16 items (*n* = 8) were more likely to use EMRs from delivery hospitalisations (100%) compared to 50% and 11% in countries with 15–14 items (*n* = 12) and < 14 items (*n* = 9), respectively. Linkage was also more common in these countries (100%) versus 75% and 56%, respectively. Other data source characteristics did not differ by the ability to provide data on core perinatal indicators.

**Conclusions:**

There are wide differences between countries in the data sources used to construct perinatal health indicators in Europe. Countries using EMR linking to other sources had the best data availability.

## 1 Background

Maternal and perinatal mortality and morbidity constitute a major societal health burden in European countries, given the long‐term health consequences for women and children and high psychological and financial costs for families and society [1]. Although mortality and morbidity during pregnancy, birth and the postpartum have declined vastly over past decades, improvements are still needed, as shown by wide variations in these outcomes between European countries [2, 3, 4, 5] and substantial socioeconomic and regional inequalities within countries [6, 7, 8]. Furthermore, in some countries, improvements in perinatal mortality have recently slowed or stopped, while others have experienced increases [9, 10, 11].

International comparisons play a critical role in raising awareness about the need and potential for change [12]. The Euro‐Peristat network, which started in 2000 to compile comparable high‐quality perinatal health indicators in Europe, has highlighted wide variation in key indicators of perinatal health [13]. Based on data collected periodically from national health information systems, large differences have been observed in rates of multiple birth, preterm birth, stillbirth, neonatal mortality, perineal tears and caesarean section rates [2, 14, 15, 16, 17]. Work by the Euro‐Peristat network has also illustrated how these data can feed into societal debates and lead to changes in policy and health [18, 19].

These international comparisons rely on the routine availability of comparable population birth data from many countries, but are constrained by the existing information systems. A first set of limits relate to the compilation of national data at an international level, such as in databases maintained by Eurostat, the European Union statistical office, the World Health Organization (WHO) or the Organisation for Economic Cooperation and Development (OECD). Perinatal data included in these databases are not always produced in a comparable manner, as evidenced by discrepancies in stillbirth rates between Euro‐Peristat and Eurostat [4]. Additionally, key indicators, such as preterm birth rates, are often missing while data are not available for subgroup analyses needed to fully understand differences, such as perinatal mortality by gestational age or socioeconomic status.

A second set of limits arises from the weaknesses of national health information systems. If data are not produced nationally, improving international collection and reporting mechanisms will not resolve the problem of data availability. The shortcomings of population birth information systems in European countries were revealed during the COVID‐19 pandemic, as the absence of key information and slow processing times impeded timely analysis of the direct and indirect impacts of the pandemic [20]. Previous work by the Euro‐Peristat project has also highlighted gaps in population birth data in Europe [21].

In order to provide guidance for improving perinatal health information systems, this study aimed to describe current population birth data sources in Europe and identify the characteristics of information systems in countries that provide data to Euro‐Peristat. We use data from the latest data collection exercise by Euro‐Peristat covering the years 2015 to 2021 which took place as part of the European Population Health Information Research Infrastructure (PHIRI) project. This project aimed to share population data using a federated model to describe the impact of the pandemic and included a use case on the indirect effects of the pandemic on perinatal health, which was undertaken by Euro‐Peristat [22].

## 2 Methods

Information about perinatal health data sources in Europe comes from the Euro‐Peristat network, which started in 2000. This network brings together 31 countries (27 EU member states plus Iceland, Norway, Switzerland, and the UK) to assess perinatal health using comparably defined perinatal health indicators constructed from routine national birth data [13]. The aim of the network is to produce high‐quality data and analysis at regular intervals for use by decision‐makers in the perinatal health sector. Network members are statisticians, data managers, epidemiologists and clinicians experienced in the use of routine health data. The network produces periodic reports [11] and publications based on these data. The network is coordinated by Inserm in Paris and has received funding from the EU's DG‐Santé and DG‐Research Agencies.

Euro‐Peristat's data collection is organised around a set of 10 core and 20 recommended indicators, selected after Delphi consensus processes with perinatal health professionals [23]. The goal was to identify a robust but constrained set of indicators in order to promote feasibility as well as comparability over time. Data are collected on all live births, fetal deaths and terminations of pregnancy (TOP) starting at 22 + 0 or more weeks of gestation, using a common protocol with harmonised definitions. Aggregated data on each indicator are compiled by relevant sub‐groups, including missing observations, so that prevalence and incidence rates can be calculated in a similar manner. For example, stillbirths and infant deaths are compiled by gestational age and multiplicity.

Data come from population‐based birth data used for monitoring perinatal health in each country. Sources include birth registers, hospital discharge data and civil registration databases. When more than one source exists, the country team decides which is best able to produce high‐quality indicators corresponding to Euro‐Peristat definitions.

Within the PHIRI project, a new federated data collection protocol was elaborated based on a proposed model for all of the use cases [24]. To test this new approach, only the core indicators were included (fetal, neonatal and infant mortality, gestational age and birthweight distributions, type of pregnancy (singleton or multiple), maternal age, parity and mode of delivery), along with recommended indicators of socioeconomic status to allow analyses of social inequalities. Maternal mortality is also a core indicator routine information system that cannot be used for comparable reporting of this rare outcome [25].

This protocol is based on the creation of a common data model at an individual‐level including data items needed to generate aggregate tables for the core indicators (16 items required). Each data provider created a database of births for the years 2015 to 2020 (updated recently to 2021), following the specifications of the common data model which defines variable names, definitions and formats. Using this individual‐level database, R programs were provided to run on each institution's server to generate aggregated anonymised national‐level data tables (see Table S1 for links to the common data model and scripts). Individual‐level data are accessed only by the statisticians or researchers with authorisations to use the data set in each country. These aggregate tables were then transferred to the central coordination office and compiled. Quality checks include verification of missing data and indicator values by data providers and analyses of consistency and external validity by the coordination team.

For the PHIRI Use Case, all data had to be in the same database to run the R scripts. Several countries carried out specific linkages of different databases for the PHIRI project, but in most countries, data came from the source used for routine perinatal surveillance. For the Nordic countries, for instance, additional registers (i.e., income and education) can be linked for specific research projects, but these linkages are not carried out routinely.

To describe the data sources used for the common data model for the years 2015 to 2021, each national partner filled in an online questionnaire administered using Lime Survey in the summer of 2023 with follow‐up to clarify responses in the autumn. The questionnaire, consisting of 28 questions, was based on a previous study done on data collected in 2004, augmented by questions on routine linkage procedures, exploratory questions about checks for data quality and use of the data sources for surveillance and research. Data providers were asked to report on the original data sources as well as the data extracted to create the common data model. Questions on inclusion criteria and coverage referred to the original data sources, not to the data exported into the common data model, since these follow standardised criteria set by the Euro‐Peristat protocol. We only included data items that could be provided in accordance with the new protocol, even though some countries provided indicators in aggregate form from other sources (i.e., neonatal and infant mortality in Italy and Spain).

Data were analysed using descriptive tables and discussed within the Euro‐Peristat network. All national contacts reviewed and amended the tables, as needed. We distinguished countries by their ability to provide the data in the common data model (all data items available [16], almost all [15–14] and incomplete [< 14]). As most countries could provide a high number of the basic data items, we chose these cut‐offs to focus on high performers providing all or almost all items.

### 2.1 Ethics Approval

Ethics approval was not required as no personal data were collected in this study on data system characteristics.

## 3 Results

**FIGURE 1:**
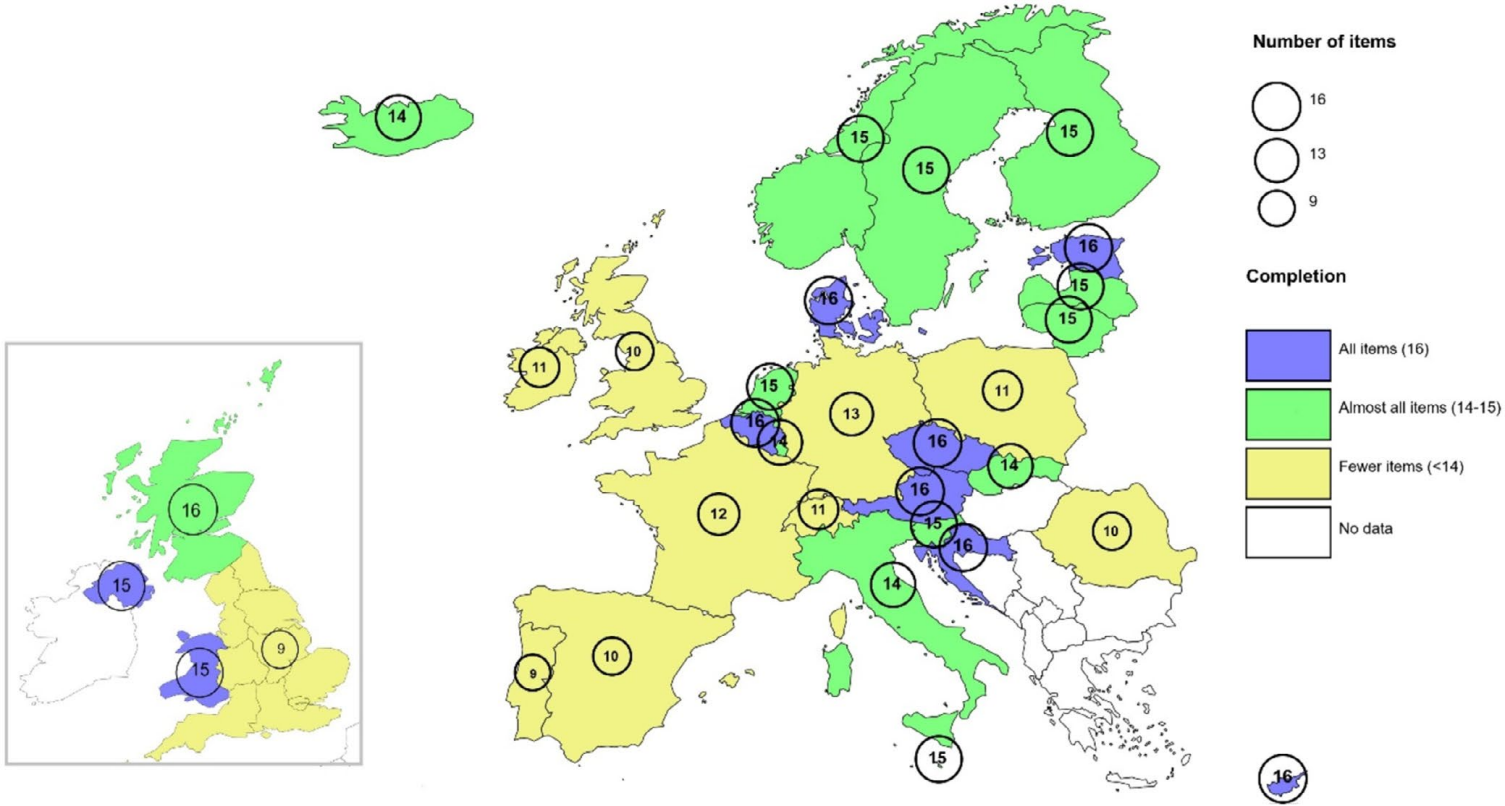
Countries participating in the Euro‐Peristat data collection and number of data items provided, out of a maximum of 16. NB: Wales provides data alone and as part of Office of National Statistics data which covers England and Wales. Romania, Slovakia and Wales did not participate in the survey on data sources.

Of the 31 countries (including the constituent nations of the UK) that created a database for the PHIRI project, 29 participated in the survey, as shown in the map in Figure 1. Table S2 provides the data sources used in each country. This map also provides information on the number of variables from the common data model that each country could provide, including non participants in the survey (Romania, Slovakia and Wales). About one‐third of countries could provide all 16 variables, with the lowest number being only 9 of the variables in Portugal. Missing information was most common for mode of delivery and neonatal and infant death (see Table S3 for missing variables by country).

Different routine sources of birth data are used to create the common data model (Table 1) including birth certificates (15 countries), electronic medical records (EMR) from delivery hospitalisations (16 countries), direct entry by health providers (9 countries), EMR from other care providers (7 countries), Hospital Discharge Summaries (7 countries) and other sources (6 countries). These other sources include national registers of persons, census or statistical data, paper records from planned out‐of‐hospital births, birth identification forms, separate notifications from the neonatal intensive care unit (NICU) containing the diagnoses for infants transferred to these units after birth and abortion and miscarriage registers.

**TABLE 1:** Sources and linkage of population birth data used to report on core perinatal health indicators in the Euro‐Peristat network.

Country (× = yes)	Birth certificates	Electronic medical records from delivery hospitalisations	Electronic medical records from other providers	Hospital discharge summaries	Direct data entry by health provider	Death certificates	Other Sources	Linkage to construct dataset	Linkage type (A: ID, B: pseudo‐ID, C: ‘other’)	Can link birth and infant death (valid)	Linkage done for Euro‐Peristat CDM
Austria	×	×	×			×		×	A	×	
Belgium	×					×	×^a^	×	C^b^	×	
Croatia		×				×		×	A	×	
Cyprus		×				×		×	C^c^	×	
Czech Republic	×	×	×		×	×		×	A	×	
Denmark		×	×	×	×		×^d^	×	B	×	
Estonia		×	×			×		×	A	×^f^	×^g^
Finland	×	×		×		×	×^e^	×	A	×^f^	×^g^
France				×		×		×	B and C^h^		×
Germany					×						
Iceland	×	×	×	×		×		×	A	×	
Ireland					×		×^i^				
Italy	×					×	×^j^				×
Latvia		×			×^k^				C^l^		
Lithuania					×^m^				C^n^		
Luxembourg					×						
Malta				×^o^		×		×	A		
Netherlands		×	×		×			×	C^b^		
Norway	×	×^p^	×				×	×	A	×	
Poland	×					×		×	C^q^	×	
Portugal	×			×		×					
Slovenia	×	×				×		×	A		
Spain	×										
Sweden		×				×		×	A and B	×	
Switzerland	×					×		×	A^r^	×	
UK‐England	×					×		×	A and B	×	
UK: N. Ireland		×		×		×		×	A	×	
UK: Scotland	×	×				×		×	A	×	
UK‐MBRRACE	×	×			×	×		×	A		
TOTAL	15	16	7	7	9	20	6	20		16	

^a^ National register of individuals.

^b^ Probabilistic.

^c^ Characteristics of the mother and the newborn.

^d^ Census/statistical data.

^e^ Paper records from planned out‐of‐hospital births.

^f^ Anonymised Ids as well as deterministic and probabilistic linkage to link mothers with babies and infant deaths with infant birth hospitalisations.

^g^ For early neonatal mortality (0–6 days) in routine.

^h^ For mortality (7 days to 1 year) done for Euro‐peristat.

^i^ Birth Notification Form (both electronic and paper).

^j^ TOP and Miscarriage Register.

^k^ Medical Birth Register.

^l^ ID, possibility of direct linkage with Causes of Death Registry, this is done by hand.

^m^ Special forms for Medical data of Births.

^n^ Done by hand.

^o^ Hospital paper medical records from delivery hospitalisations.

^p^ We receive a separate notification from the NICU containing the diagnoses for infants transferred to these units after birth.

^q^ Own key.

^r^ Data linkage by pseudo‐ID between the Medical statistics of hospitals (MS) and the civil registry sources (BEVNAT) will be possible in 2024.

Most countries combine more than one data source for routine surveillance of perinatal health, with six countries using four or five different sources. However, six countries (Germany, Ireland, Latvia, Luxembourg, Lithuania and Spain) use a single source. In these countries, the source is direct entry by health care providers, with the exception of Spain which uses only birth certificate data. 15 of the 20 countries that link data sources use a personal identifier while four use a pseudo‐identifier (a single pseudonym replaces the identifier for linkage). In a few countries, linkage is not done routinely, but was implemented to create the common data model for the Euro‐Peristat data collection exercise (Estonia, France and Italy). Linkage between birth records and infant death certificates is only done in 16 countries.

Reporting is obligatory in all countries (Table 2). The databases most often include mothers giving birth in the country, regardless of their nationality or place of residence (24 countries). Some countries only include births to residents (Austria, Poland). Some sources also include births to citizens occurring abroad (Belgium, Czech Republic, Denmark, Portugal, Spain and Switzerland). Completeness of coverage is very good, with 17 countries covering all births and the others reporting coverage rates of over 98%. One reason for incomplete coverage is use of hospital discharge data, as seen in France, where home births are not included unless there is a subsequent hospitalisation of the mother or baby. Other reasons are related to incomplete recording of stillbirths or terminations.

**TABLE 2:** Coverage and completeness of the data sources used to report on core perinatal health indicators in the Euro‐Peristat network.

Country	Participation in database	Completeness	Population coverage			
			Citizens who are residents	Citizens delivering out of the country	Non citizens who are residents	Noncitizens nonresidents
Austria	Obligatory	100%	×		×	
Belgium	Obligatory	~100%	×	×^a^	×	×
Croatia	Obligatory	> 98%	×		×	×
Cyprus	Obligatory	99.9%	×		×	×
Czech Republic	Obligatory	100%	×	×	×	
Denmark	Obligatory	100%	×	×	×	×
Estonia	Obligatory	100%	×		×	×
Finland	Obligatory	100%	×		×	×
France	Obligatory	99%^b^	×		×	×
Germany	Obligatory	98%	×		×	×
Iceland^b^	Obligatory	100%	×		×	×
Ireland	Obligatory	100%	×		×	×
Italy	Obligatory	Live births and stillbirths ≥ 26 weeks: 100% Stillbirths < 26 weeks: 95% TOPS: 97%	×		×	×
Latvia	Obligatory	~99%^c^	×		×	×
Lithuania	Obligatory	~99%^d^	×		×	×
Luxembourg	Obligatory^e^	~99%^f^	×		×	×
Malta	Obligatory	100%	×		×	×
Netherlands	Obligatory	97%–98%	×		×	×
Norway	Obligatory	~100%	×		×	×
Poland	Obligatory	100%	×		×	
Portugal	Obligatory	100%	×	×	×	×
Slovenia	Obligatory	100%	×		×	×
Spain	Obligatory	100%	×	×	×	
Sweden	Obligatory	98%	×		×	×
Switzerland	Obligatory	> 98%	×	×	×	
UK‐ England and Wales	Obligatory	100%	×		×	×
UK: N. Ireland	Obligatory	100%	×		×	×
UK: Scotland	Obligatory	Births 100%, hospital records^g^ 98%–99%, TOPs underestimated	×		×	×
UK‐MBRRACE	Obligatory	Terminations are identified but excluded	×		×	×

^a^ The Belgian databases include births to residents abroad, but for these we only have data from the National Register (sociodemographic data: date of birth, country of birth, nationality, multiple births, data on the mother if she is a resident: date of birth, nationality, marital status, etc.).

^b^ Births out of the hospital that do not come to the hospital are not included.

^c^ Births born outside of the country are not included.

^d^ The Medical data of Birth covers newborns and stillbirths born in maternal hospitals of Lithuania, including foreign citizens, but they not include Lithuanian citizens and residents born outside country.

^e^ Tacit participation of the individuals in the perinatal register, with possibility to refuse collection of additional data beyond basic birth details.

^f^ From 2021, home births are not exhaustive.

^g^ Scottish Morbidity Record 02 (an episode‐based patient record relating to all inpatients and day cases discharged from Obstetric specialities in the NHS Scotland).

In the routine databases used to build the common data model for the federated analysis, gestational age and birthweight inclusion criteria vary (Table 3). The majority of countries have no limits on either gestational age or birthweight for live birth registration (21 countries). Other countries have specified inclusion thresholds: ≥ 22 weeks (Iceland), ≥ 22 weeks or ≥ 500 g (France, Italy, Norway, Spain), ≥ 20 weeks or ≥ 400 g (UK, Mothers and Babies: Reducing Risk through Audits and Confidential Enquiries [MBRRACE] data) and ≥ 500 g (Portugal). For stillbirths, inclusion criteria vary more widely: 10 countries use the criterion ≥ 22 weeks or ≥ 500 g and 11 countries use the criterion of ≥ 22 weeks of gestational age and do not include births over 500 g if they are under 22 weeks. Four countries use a lower threshold based on a birthweight of 500 g: Austria, Belgium, Germany and Poland. Ireland uses a specific criterion of ≥ 24 weeks or ≥ 500 g, while Portugal, England and Wales and Scotland use ≥ 24 weeks. In Italy, registration of fetal deaths as stillbirths starts at 180 days of gestation (25 weeks + 5 days), although data on stillbirths under this limit are obtained for Euro‐Peristat by linking with databases recording early losses. Scotland can also add abortions at < 24 weeks.

**TABLE 3:** Gestational age and birthweight thresholds for recording stillbirths and live births and inclusion of terminations of pregnancy (TOP) in the data sources used for the Euro‐Peristat common data model.

Country	Stillbirths	Live births	Cannot provide Euro‐Peristat criteria^a^	TOP ≥ 22 weeks in the data source	Able to differentiate TOP from stillbirths
Austria^b^	≥ 500 g	No limit	×		
Belgium	≥ 500 g	No limit	×	×	
Croatia	≥ 22 weeks or ≥ 500 g	No limit		×	
Cyprus	≥ 22 weeks or ≥ 500 g	No limit		×	×
Czech Republic	≥ 22 weeks or ≥ 500 g	No limit		×	
Denmark	≥ 22 weeks	No limit		×	×
Estonia	≥ 22 weeks	No limit			N/A^c^
Finland	≥ 22 weeks or ≥ 500 g	No limit		×^d^	×
France	≥ 22 weeks or ≥ 500 g	≥ 22 weeks or ≥ 500 g		×	×
Germany	≥ 500 g or ≥ 23 weeks	No limit	×		N/A
Iceland	≥ 22 weeks	≥ 22 weeks			N/A
Ireland	≥ 24 weeks or ≥ 500 g	No limit	×	×^e^	
Italy	≥ 22 weeks^f^	≥ 22 weeks or ≥ 500 g		×	×
Latvia	≥ 22 weeks	≥ 22 weeks		×	×
Lithuania	≥ 22 weeks	No limit			N/A
Luxembourg	≥ 22 weeks or ≥ 500 g	No limit		×	×
Malta	≥ 22 weeks	No limit		N/A^g^	N/A
Netherlands	≥ 22 weeks or ≥ 500 g	No limit		×^h^	×^h^
Norway	≥ 22 weeks or ≥ 500 g	≥ 22 weeks or ≥ 500 g		×^i^	×
Poland	≥ 500 g	No limit	×		N/A
Portugal	≥ 24 weeks	≥ 500 g	×		
Slovenia	≥ 22 weeks	No limit		×	×
Spain	≥ 22 weeks or ≥ 500 g	≥ 22 weeks or ≥ 500 g			N/A
Sweden	≥ 22 weeks	No limit			N/A
Switzerland	≥ 22 weeks or ≥ 500 g	No limit		×	×^j^
UK‐England and Wales	≥ 24 weeks	No limit		×	×
UK: Northern Ireland	≥ 22 weeks	No limit		×	
UK: Scotland	If ≥ 24 weeks code as stillbirth. If < 24 weeks code as an abortion episode^k^	No limit		×	×
UK‐MBRRACE	≥ 22 weeks	≥ 20 weeks or ≥ 400 g			N/A

^a^ ≥ 22 weeks, if GA unknown ≥ 500 g.

^b^ Austria, TOPS ending in a live birth are included.

^c^ Until 2020 separate registers for TOP and births (including stillbirths), from 2021 onwards in one register. TOP is allowed until 21 weeks and 6 days and registered in Abortion Register until 2020.

^d^ TOP is possible until 24 + 0 weeks, but these are not reported to the Medical Birth Register (but to Register on Induced Abortions).

^e^ A small number of records of TOPs that are ≥ 24 weeks or ≥ 500 g are included on the file, but we don't differentiate them from stillbirths.

^f^ Stillbirths ≥ 180 days in birth certificates and stillbirths < 180 days from the miscarriage register.

^g^ TOPs are illegal.

^h^ Yes, however likely underreported.

^i^ Only terminations of pregnancy for fetal anomalies (TOPFA).

^j^ Possible to link TOPs from Causes of death and births so information can be found.

^k^ Spontaneous and induced abortions in an obstetric setting.

Seventeen countries include TOP in routine birth data (Table 3). Among these 18 countries, 12 are able to differentiate between TOP and spontaneous stillbirths. There are several reasons for not including TOP: TOP are not legal or the legal limit is before 22 weeks, TOP at or after 22 weeks are rare, or the existence of separate TOP registers.

The responses to exploratory questions about data quality are presented in Table S4. These show that while most systems include checks for data quality, the methods employed are diverse and complex to summarise. In many systems, the data are checked, and data providers are contacted about missing variables or outlier values, but countries differ in terms of variables analysed and criteria for data verification. These checks can be done at different levels, hospital, regional and national and most can recontact the data providers. Twenty‐one countries reported that they have the capacity to correct or update data, with 13 stating that the time frame for these updates or corrections was not limited.

These data are an important source for scientific research: All countries declared that these data are used for research, 24 countries out of the 27 have an annual report (Table S5) and some examples of research publications are given in Table S6.

Table 4 links the characteristics of the data sources to the countries' ability to provide data for the common data model. Countries providing all data were more likely to use EMRs from delivery hospitalisations, 100%, compared to 50% and 11% in the other countries providing fewer variables. Linkage was also more common in these countries (100%), followed by the countries providing almost all variables (75%) and countries providing few variables (56%). Countries who provide all data routinely link birth and death certificates (100%), whereas this proportion is lower (42% and 33%) for countries who could not provide all the variables. The other data source characteristics did not differ by the ability to provide data for the common data model.

**TABLE 4:** Link between data source characteristics and the ability to provide data items core perinatal health indicators in the Euro‐Peristat common data model.

	All items 16/16 (*N* = 8)	Almost all items 14–15/16 (*N* = 12)	Fewer items 10–13/16 (*N* = 9)
Type of data source			
Birth certificates	4 (50%)	5 (42%)	6 (67%)
EMR delivery hospitalisations	8 (100%)	6 (50%)	1 (11%)
EMR other sources	5 (63%)	2 (17%)	1 (11%)
Hospital discharge summaries	1 (13%)	4 (33%)	1 (11%)
Death certificates	6 (75%)	8 (67%)	5 (56%)
Direct data entry by health provider	2 (25%)	4 (33%)	3 (33%)
Linkage (any)	8 (100%)	9 (75%)	5 (56%)
Can link infant mortality	8 (100%)	5 (42%)	3 (33%)
Linkage methods			
Done with IDs	5 (62%)	7 (58%)	3 (33%)
Pseudo‐IDs	1 (12%)	2 (17%)	2 (22%)
Other methods	2 (25%)	2 (17%)	2 (22%)
Euro‐Peristat inclusion criteria			
Yes, live births	8 (100%)	12 (100%)	8 (100%)
Yes, stillbirths	6 (75%)	12 (100%)	5 (56%)
Able to differentiate TOP from SB	3 (38%)	7 (58%)	3 (33%)

## 4 Comment

### 4.1 Principal Findings

In Europe, the data sources used for perinatal health monitoring vary by country, ranging from civil registration data, hospital data and EMR. Countries using EMR were more likely to provide all of the 16 items included in Euro‐Peristat's Core common data model. Another feature of health information systems that were able to provide more data items was routine linkage between data sources. While most countries have 100% coverage and report on all births occurring in the country, in a small number of countries the system only includes legal residents, while systems based on hospital data do not include out‐of‐hospital births unless the mother and/or the baby are hospitalised after birth. Finally, differences in inclusion criteria for live and stillbirths and TOP remain, although the majority of countries can provide births according to the Euro‐Peristat inclusion criteria of ≥ 22 weeks of gestation. Most of those that cannot provide the Euro‐Peristat inclusion criteria use a lower limit of 500 g for stillbirths which leads to omission of stillbirths ≥ 22 weeks of gestation with birthweights < 500 g.

### 4.2 Strengths of the Study

The study builds on a longstanding research network to provide a currently unavailable overview of population data on births in European countries.

### 4.3 Limitations of the Data

The absence of validation studies for most sources limits comparison of data quality.

### 4.4 Interpretation

The previous Euro‐Peristat methodological paper [21], which described data systems in 2004, highlighted six major obstacles for perinatal health monitoring: differences in registration criteria, incomplete coverage of data collection, use of non standard definitions, variation in denominators and numerators, different ways of handling missing data and random variation in rare events, especially for countries with small populations. This updated data survey showed an improved situation for some of these obstacles linked to data availability, such as fewer countries that cannot comply with Euro‐Peristat inclusion criteria. This trend is consistent with better reporting at low gestational ages for stillbirths that has been observed in successive Euro‐Peristat studies [26, 27]. Some countries improved their coverage, for instance Italy and Denmark, where reported rates of coverage are higher than in the previous survey. There are also a few more countries that can provide national data on births, such as France, where the use of hospital discharge data within the French National Health Data System makes it possible to provide perinatal variables [28] as well as Cyprus which did not register stillbirths at the time of the first study. Furthermore, the new protocol for data collection, which replaced the collection of provider‐generated aggregated Excel tables, has addressed obstacles related to non standard definitions, different denominators and numerators when data are derived from different sources or because of missing data [24]. Due to the structure of the common data model, it was necessary to specify missing data for each variable, making it easier to collect this information.

Despite this progress, however, challenges in providing data persist almost 20 years after the previous review, as seen by the high number of countries unable to provide all variables for creating a core set of perinatal health indicators. Furthermore, the common data model adds the constraint that all data items need to be contained in the same data source. The absence of key data items shows that many countries still do not carry out basic linkage between routine sources [29], such as information on births with death certificates—to derive neonatal and infant mortality—or information on births with hospital data in order to derive caesarean section rates, for instance in Switzerland, UK‐England, UK‐MBRRACE, Poland, Portugal and Romania. Some countries, such as Sweden and Norway did not provide data on socioeconomic status, even though these data exist in other registers, because they are not routinely linked and therefore not readily available for surveillance and monitoring purposes. Finally, data quality is difficult to evaluate. Exploratory questions on quality checks yielded diverse responses, revealing the need for work to standardise indicators of quality across data systems. Only a few countries have published reports and validation studies [30, 31] and this is an area for future work. Validation studies are also important in order to understand the impact of diversity in type of source (hospital data versus EMR) and coverage (citizens or residents versus all births in the country).

In the era of big data, it is surprising that high‐income countries still struggle to provide basic population‐level information on the characteristics of the childbearing and infant populations and indicators of their health. While there are a growing number of initiatives to improve data through better procedures for linkage, these are often only carried out for research projects [32] and not scaled up or maintained on a regular basis to facilitate routine reporting. Yet efforts to create ‘research ready’ data [33] should also be justified because they allow robust surveillance data and cross‐country comparisons.

Our finding that systems based on EMR were more likely to provide all data items may reflect the greater breadth and flexibility of these systems in comparison with vital statistics or hospital discharge data and suggest that integration of EMR with these other systems could be an effective improvement strategy. Other initiatives to improve perinatal data collection have led to more comprehensive information systems at the regional level, especially in larger countries with decentralised health information systems, such as Spain or Italy. For instance, in Spain, some Autonomous Regions routinely link birth and death certificate data. These initiatives can provide tested blueprints to inform health information policies, but need to be generalised nationally. The big data era may create a false sense of assurance, leading to less willingness to finance data systems and quality programmes despite the need for strategic public investment. It is important to communicate with policymakers about the value of high‐quality information systems and the need to improve the quality in addition to the quantity of data [34]. These investments in routine health data are also needed for research, as shown by the use of the Nordic registers for knowledge generation in perinatal health [35], as well as projects such as PHIRI and others to establish federated platforms using these sources. The ConcePTION project is one such project, for instance, creating a platform for the real‐world investigation of medications during pregnancy and breastfeeding [36, 37].

### 4.5 Conclusions

Despite some progress over past decades, further improvements in national data sources are needed before most countries in Europe can provide key indicators on the health of pregnant women and babies. Linkage of data sources and use of EMR for routine reporting were more common in countries that had more indicators available, but other data sources were also used to achieve good data availability. This study demonstrates the need to raise awareness about the limits of current systems and the benefits of having a high‐quality perinatal health information infrastructure in many countries.

## Author Contributions

M.P. and J.Z. conceived and planned the present work. Data were collected by M.G., O.Z., S.R., Z.D., G.H., A.M., A.M., L.S., V.T., P.V., J.K., L.B. and T.R. and data collection was coordinated by J.Z. and M.P. M.P. carried out the analysis. M.P. drafted the initial manuscript, with support from J.Z. and M.G. All authors, including those in Euro‐Peristat Group Author, participated in the study design and data collection for their countries and reviewed, edited and approved the final draft.

## Conflicts of Interest

The authors declare no conflicts of interest.

## Supporting information


Data S1.

## Data Availability

All data from the survey of data providers are published in the article and in the Supporting Information.

## Appendix A The Euro‐Peristat Research Group

Austria—Alex Farr (The Medical University of Vienna, Department of Obstetrics and Gynaecology, Vienna), Jeannette Klimont (Statistics Austria, Vienna); Belgium—Sophie Alexander, Wei‐Hong Zhang (Perinatal Epidemiology and Reproductive Health Unit, CR2, School of Public Health, ULB, Brussels), Gisèle Vandervelpen (Statbel, Brussels); Bulgaria—Rumyana Kolarova (Directorate Budget and Finance, Ministry of Health, Sofia), Evelin Yordanova (Statistics of health and justice, National Statistical Institute, Sofia); Croatia—Željka Draušnik (Croatian Institute of Public Health, Zagreb); Cyprus—Theopisti Kyprianou, Vasos Scoutellas (Health Monitoring Unit, Ministry of Health, Nicosia); Czech Republic—Petr Velebil (Institute for the Care of Mother and Child, Prague); Denmark—Laust Hvas Mortensen (Department of Public Health, University of Copenhagen, Copenhagen and Denmark Statistics, Copenhagen); Estonia—Luule Sakkeus, Liili Abuladze (Estonian Institute for Population Studies, Tallinn University, Tallinn); Finland—Mika Gissler (THL Finnish Institute for Health and Welfare, Information Services Department, Helsinki and Karolinska Institute, Department of Neurobiology, Care Sciences and Society, Stockholm), Anna Heino (THL Finnish Institute for Health and Welfare, Helsinki); France—Béatrice Blondel, Anne Chantry, Catherine Deneux‐Tharaux, Mélanie Durox, Alice Hocquette, Marianne Philibert, Jennifer Zeitlin (Université de Paris, CRESS, Obstetrical Perinatal and Paediatric Epidemiology Research Team, EPOPé, INSERM, INRA, Paris), Jeanne Fresson (Population Health Office, Directorate of Research, Study, Evaluation and Statistics (DREES), Health Ministry, Paris); Germany—Guenther Heller (Institute for Quality Assurance and Transparency in Healthcare IQTIG, Berlin), Björn Misselwitz (Institute of Quality Assurance Hesse, Eschborn); Greece—Aris Antsaklis (IASO Maternity Hospital, Department of Fetal Maternal and Perinatal Medicine, University of Athens, Athens); Hungary—István Sziller (National Directory for Hospital Management, Budapest); Iceland—Johanna Gunnarsdóttir (Landspitali University Hospital, Reykjavik), Helga Sól Ólafsdóttir (Departement of Obstetrics and Gynaecology, Landspitali University Hospital, Reykjavik); Ireland—Karen Kearns (Healthcare Pricing Office, National Finance Division, HSE, Dublin), Izabela Sikora (The National Perinatal Reporting System, Health Pricing Office, Dublin); Italy—Marina Cuttini (IRCCS—Associazione La Nostra Famiglia, Istituto Scientifico Eugenio Medea, Bosisio‐Parini, Lecco), Marzia Loghi and Marilena Pappagallo (Directorate for Social Statistics and Welfare, Italian Statistical Institute (ISTAT), Rome), Serena Donati (National Centre for Disease Prevention and Health promotion, Istituto Siperiore di Sanità, Italian National Health, Rome), Rosarlia Boldrini (General Directorate for the Health Information and Statistical System, Italian Ministry of Health, Rome); Latvia—Janis Misins, Irisa Zile (The Centre for Disease Prevention and Control of Latvia, Riga); Lithuania—Jelena Isakova (Institute of Hygiene, Health Information Centre, Health Statistics Department, Vilnius); Luxembourg—Aline Lecomte, Audrey Billy, Jessica Pastore (Department of Precision Health, Luxembourg Institute of Health, Luxembourg), Daniel Alvarez (Department of Epidemiology and Statistics, Directorate of Health, Luxembourg); Malta—Miriam Gatt (Directorate for Health Information and Research, National Obstetric Information Systems (NOIS) Register, Tal‐Pietà); Netherlands—Jan Nijhuis (Department of Obstetrics & Gynaecology, Maastricht University Medical Centre, MUMC+, Maastricht), Lisa Broeders (The Netherlands Perinatal Registry (Perined), Utrecht), Peter Achterberg (National Institute for Public Health and the Environment, Bilthoven), Ashna Hindori‐Mohangoo (Foundation for Perinatal Interventions and Research in Suriname (PeriSur), Paramaribo, Suriname); Norway—Kari Klungsoyr (Division of Mental and Physical Health, Norwegian Institute of Public Health, Bergen, Norway and Department of Global Public Health and Primary Care, University of Bergen), Rupali Akerkar, Hilde Engjom (Health Registry Research and Development, Norwegian Institute of Public Health, Bergen); Poland—Katarzyna Szamotulska, Ewa Mierzejewska (Department of Epidemiology and Biostatistics, National Research Institute of Mother and Child, Warsaw); Portugal—Henrique Barros (University of Porto Medical School, Department of Public Health, Forensic Sciences and Medical Education, Porto); Romania—Mihai Horga (East European Institute for Reproductive Health, Târgu Mureş), Vlad Tica (East European Institute for Reproductive Health, Faculty of Medicine, University “Ovidius”, Constanţa); Lucian Puscasiu (East European Institute for Reproductive Health, University of Medicine, Pharmacy, Science and Technology “George Emil Palade”, Târgu Mureş), Mihaela‐Alexandra Budianu (Obstetrics and Gynaecology Clinic, University of Medicine and Pharmacy, Târgu Mureş), Alexandra Cucu (National Centre for Noncommunicable Diseases Surveillance, National Institute of Public Health, Bucharest), Cristian Calomfirescu (National Center for Statistics in Public Health, National Institute of Public Health, Bucharest); Slovakia—Jan Cap (National Health Information Center, Bratislava); Slovenia—Miha Lučovnik, Ivan Verdenik (University Medical Centre, Department of Obstetrics & Gynaecology, Ljubljana); Spain—Oscar Zurriaga (Preventive Medicine and Public Health Departament, University of Valencia and FISABIO, Rare Diseases Research Area and Mixed Unit FISABIO‐UVEG, Valencia), Adela Recio Alcaide (National Institute for Statistics (INE), Madrid), María Fernández‐Elorriaga (Autonomous University, Madrid), Mireia Jané (Public Health Surveillance Direction, Catalan Public Health Agency Generalitat de Catalunya, Barcelona), Maria José Vidal (Public Health Surveillance Direction, Catalan Public Health Agency Generalitat de Catalunya, Barcelona); Sweden—Karin Källén, Anastasia Nyman (The National Board of Health and Welfare, Department of Evaluation and Analysis, Epidemiology and Methodological Support Unit, Stockholm); Switzerland—Tonia Rihs (Federal Statistical Office FSO, Neuchâtel); United Kingdom—Alison Macfarlane (Centre for Maternal and Child Health Research, School of Health Sciences, City University of London, London), Sonya Scott, Kirsten Monteath (Public Health Scotland, Edinburgh), Lucy Smith, Ruth Matthews (Department of Population Health Sciences, College of Life Sciences, University of Leicester, Leicester), Siobhán Morgan (Hospital Information Branch, Department of Health, Stormont Estate, Belfast), Joanne Murphy(Public Health Agency, Belfast).

## References

[1] J. Zeitlin , B. Blondel , and B. Khoshnood , “Fertility, Pregnancy and Childbirth,” in Successes and Failures of Health Policy in Europe Over Four Decades: Diverging Trends, Converging Challenges. European Observatory on Health Systems and Policies, ed. J. M. M. Mackenbach (Berkshire, England: Open University Press McGraw‐Hill, 2013).

[2] B. Blondel , M. Cuttini , A. D. Hindori‐Mohangoo , et al., “How Do Late Terminations of Pregnancy Affect Comparisons of Stillbirth Rates in Europe? Analyses of Aggregated Routine Data From the Euro‐Peristat Project,” British Journal of Obstetrics and Gynaecology 125, no. 2 (2018): 226–234, 10.1111/1471-0528.14767. 28557289

[3] J. Zeitlin , L. Mortensen , M. Cuttini , et al., “Declines in Stillbirth and Neonatal Mortality Rates in Europe Between 2004 and 2010: Results From the Euro‐Peristat Project,” Journal of Epidemiology and Community Health 70, no. 6 (2016): 609–615, 10.1136/jech-2015-207013. 26719590 PMC4893141

[4] C. Diguisto , M. Saucedo , A. Kallianidis , et al., “Maternal Mortality in Eight European Countries With Enhanced Surveillance Systems: Descriptive Population Based Study,” British Medical Journal 379 (2022): e070621, 10.1136/bmj-2022-070621. 36384872 PMC9667469

[5] K. Helenius , G. Sjors , P. S. Shah , et al., “Survival in Very Preterm Infants: An International Comparison of 10 National Neonatal Networks,” Pediatrics 140, no. 6 (2017): e20171264, 10.1542/peds.2017-1264. 29162660

[6] J. Zeitlin , L. Mortensen , C. Prunet , et al., “Socioeconomic Inequalities in Stillbirth Rates in Europe: Measuring the Gap Using Routine Data From the Euro‐Peristat Project,” BMC Pregnancy and Childbirth 16 (2016): 15, 10.1186/s12884-016-0804-4. 26809989 PMC4727282

[7] J. Jardine , K. Walker , I. Gurol‐Urganci , et al., “Adverse Pregnancy Outcomes Attributable to Socioeconomic and Ethnic Inequalities in England: A National Cohort Study,” Lancet 398, no. 10314 (2021): 1905–1912, 10.1016/S0140-6736(21)01595-6. 34735797

[8] G. Poulsen , K. Strandberg‐Larsen , L. Mortensen , et al., “Exploring Educational Disparities in Risk of Preterm Delivery: A Comparative Study of 12 European Birth Cohorts,” Paediatric and Perinatal Epidemiology 29, no. 3 (2015): 172–183, 10.1111/ppe.12185. 25808200

[9] G. Heller , S. Schill , and D. Münch , “Früh‐ Und Totgeburtlichkeit im Zeitlichen Und Internationalen Kontext,” (2023), https://www.g‐ba.de/downloads/17‐98‐5617/PV4_1_Heller_Frueh‐und‐Totgeburtlichkeit_2023‐11‐24.pdf.

[10] N. T. H. Trinh , S. de Visme , J. F. Cohen , et al., “Recent Historic Increase of Infant Mortality in France: A Time‐Series Analysis, 2001 to 2019,” Lancet Regional Health Europe 16 (2022): 100339, 10.1016/j.lanepe.2022.100339. 35252944 PMC8891691

[11] Euro‐Peristat project , “European Perinatal Health Report,” (2022), Core Indicators of the Health and Care of Pregnant Women and Babies in Europe From 2015 to 2019.

[12] A. Fehr , C. Lange , J. Fuchs , H. Neuhauser , and R. Schmitz , “Health Monitoring and Health Indicators in Europe,” Journal of Health Monitoring 2, no. 1 (2017): 3–21, 10.17886/RKI-GBE-2017-020.2. PMC10161272 37151308

[13] J. Zeitlin , S. Alexander , H. Barros , et al., “Perinatal Health Monitoring Through a European Lens: Eight Lessons From the Euro‐Peristat Report on 2015 Births,” British Journal of Obstetrics and Gynaecology 126, no. 13 (2019): 1518–1522, 10.1111/1471-0528.15857. 31260601

[14] A. J. Macfarlane , B. Blondel , A. D. Mohangoo , et al., “Wide Differences in Mode of Delivery Within Europe: Risk‐Stratified Analyses of Aggregated Routine Data From the Euro‐Peristat Study,” British Journal of Obstetrics and Gynaecology 123, no. 4 (2016): 559–568, 10.1111/1471-0528.13284. 25753683

[15] B. Blondel , S. Alexander , R. I. Bjarnadottir , et al., “Variations in Rates of Severe Perineal Tears and Episiotomies in 20 European Countries: A Study Based on Routine National Data in Euro‐Peristat Project,” Acta Obstetricia et Gynecologica Scandinavica 95, no. 7 (2016): 746–754, 10.1111/aogs.12894. 26958827

[16] M. Delnord , A. D. Hindori‐Mohangoo , L. K. Smith , et al., “Variations in Very Preterm Birth Rates in 30 High‐Income Countries: Are Valid International Comparisons Possible Using Routine Data?,” British Journal of Obstetrics and Gynaecology 124, no. 5 (2017): 785–794, 10.1111/1471-0528.14273. 27613083 PMC5346062

[17] A. Heino , M. Gissler , A. D. Hindori‐Mohangoo , et al., “Variations in Multiple Birth Rates and Impact on Perinatal Outcomes in Europe,” PLoS One 11, no. 3 (2016): e0149252, 10.1371/journal.pone.0149252. 26930069 PMC4773186

[18] A. D. Mohangoo , S. E. Buitendijk , C. W. Hukkelhoven , et al., “Higher Perinatal Mortality in The Netherlands Than in Other European Countries: The Peristat‐II Study,” Nederlands Tijdschrift Voor Geneeskunde 152, no. 50 (2008): 2718–2727. 19192585

[19] B. Blondel , M. Durox , and J. Zeitlin , “How Perinatal Health in France Compared With Other European Countries in 2015: Some Progress but Also Some Concerns About Newborn Health,” Journal of Gynecology, Obstetrics, and Human Reproduction 48, no. 7 (2019): 437–439, 10.1016/j.jogoh.2019.01.013. 30690087

[20] Euro‐Peristat Research N , “Population Birth Data and Pandemic Readiness in Europe,” British Journal of Obstetrics and Gynaecology 129, no. 2 (2021): 179–184, 10.1111/1471-0528.16946. 34569700 PMC8652502

[21] M. Gissler , A. D. Mohangoo , B. Blondel , et al., “Perinatal Health Monitoring in Europe: Results From the EURO‐PERISTAT Project,” Informatics for Health & Social Care 35, no. 2 (2010): 64–79, 10.3109/17538157.2010.492923. 20726736

[22] J. Zeitlin , M. Philibert , F. Estupiñán‐Romero , et al., “Developing and testing a protocol using a common data model for federated collection and analysis of national perinatal health indicators in Europe [version 2; peer review: 2 approved],” Open Res Europe 3 (2023): 54, 10.12688/openreseurope.15701.2. PMC10565425 37830050

[23] J. Zeitlin , K. Wildman , G. Breart , et al., “PERISTAT: Indicators for Monitoring and Evaluating Perinatal Health in Europe,” European Journal of Public Health 13, no. 3 Suppl (2003): 29–37. 14533746 10.1093/eurpub/13.suppl_1.29

[24] J. Zeitlin , M. Philibert , F. Estupiñán‐Romero , et al., “Developing and Testing a Protocol Using a Common Data Model for Federated Collection and Analysis of National Perinatal Health Indicators in Europe,” Open Research Europe 3 (2023): 54, https://open‐research‐europe.ec.europa.eu/articles/3‐54. 37830050 10.12688/openreseurope.15701.2 PMC10565425

[25] M. H. Bouvier‐Colle , A. D. Mohangoo , M. Gissler , et al., “What About the Mothers? An Analysis of Maternal Mortality and Morbidity in Perinatal Health Surveillance Systems in Europe,” British Journal of Obstetrics and Gynaecology 119, no. 7 (2012): 880–889, 10.1111/j.1471-0528.2012.03330.x. 22571748 PMC3472023

[26] A. D. Mohangoo , B. Blondel , M. Gissler , et al., “International Comparisons of Fetal and Neonatal Mortality Rates in High‐Income Countries: Should Exclusion Thresholds Be Based on Birth Weight or Gestational Age?,” PLoS One 8, no. 5 (2013): e64869, 10.1371/journal.pone.0064869. 23700489 PMC3658983

[27] L. K. Smith , A. D. Hindori‐Mohangoo , M. Delnord , et al., “Quantifying the Burden of Stillbirths Before 28 Weeks of Completed Gestational Age in High‐Income Countries: A Population‐Based Study of 19 European Countries,” Lancet 392, no. 10158 (2018): 1639–1646, 10.1016/S0140-6736(18)31651-9. 30269877

[28] P. Tuppin , J. Rudant , P. Constantinou , et al., “Value of a National Administrative Database to Guide Public Decisions: From the Systeme National D'information Interregimes de l'Assurance Maladie (SNIIRAM) to the Systeme National Des Donnees de Sante (SNDS) in France,” Revue D'epidemiologie et de Sante Publique 65, no. Suppl 4 (2017): S149–S167, 10.1016/j.respe.2017.05.004. 28756037

[29] M. Delnord , K. Szamotulska , A. D. Hindori‐Mohangoo , et al., “Linking Databases on Perinatal Health: A Review of the Literature and Current Practices in Europe,” European Journal of Public Health 26, no. 3 (2016): 422–430, 10.1093/eurpub/ckv231. 26891058 PMC4884328

[30] M. Bliddal , A. Broe , A. Pottegard , J. Olsen , and J. Langhoff‐Roos , “The Danish Medical Birth Register,” European Journal of Epidemiology 33, no. 1 (2018): 27–36, 10.1007/s10654-018-0356-1. 29349587

[31] S. Cnattingius , K. Kallen , A. Sandstrom , et al., “The Swedish Medical Birth Register During Five Decades: Documentation of the Content and Quality of the Register,” European Journal of Epidemiology 38, no. 1 (2023): 109–120, 10.1007/s10654-022-00947-5. 36595114 PMC9867659

[32] N. Dattani and A. Macfarlane , “Linkage of Maternity Hospital Episode Statistics Data to Birth Registration and Notification Records for Births in England 2005–2014: Methods. A Population‐Based Birth Cohort Study,” BMJ Open 8, no. 2 (2018): e017897, 10.1136/bmjopen-2017-017897. PMC5829879 29449289

[33] L. M. Grath‐Lone , M. A. Jay , R. Blackburn , et al., “What Makes Administrative Data ‘Research‐Ready’? A Systematic Review and Thematic Analysis of Published Literature,” International Journal of Population Data Science 7, no. 1 (2022): 1718, 10.23889/ijpds.v6i1.1718. 35520099 PMC9052961

[34] R. M. Kaplan , D. A. Chambers , and R. E. Glasgow , “Big Data and Large Sample Size: A Cautionary Note on the Potential for Bias,” Clinical and Translational Science 7, no. 4 (2014): 342–346, 10.1111/cts.12178. 25043853 PMC5439816

[35] K. Laugesen , J. F. Ludvigsson , M. Schmidt , et al., “Nordic Health Registry‐Based Research: A Review of Health Care Systems and Key Registries,” Clinical Epidemiology 13 (2021): 533–554, 10.2147/CLEP.S314959. 34321928 PMC8302231

[36] R. Pajouheshnia , R. Gini , L. Gutierrez , et al., “Metadata for Data dIscoverability aNd Study rEplicability in obseRVAtional Studies (MINERVA): Development and Pilot of a Metadata List and Catalogue in Europe,” Pharmacoepidemiology and Drug Safety 33, no. 8 (2024): e5871, 10.1002/pds.5871. 39145406

[37] N. H. Thurin , R. Pajouheshnia , G. Roberto , et al., “From Inception to ConcePTION: Genesis of a Network to Support Better Monitoring and Communication of Medication Safety During Pregnancy and Breastfeeding,” Clinical Pharmacology and Therapeutics 111, no. 1 (2022): 321–331, 10.1002/cpt.2476. 34826340 PMC9299060

