# Supplementary material for: Evaluating Perinatal Health in Europe: A Comparison of Routine Population Birth Data Sources
Source: Paediatr Perinat Epidemiol. 2025 Mar 11;39(5):405–16. doi: 10.1111/ppe.13178 (PMC12308632; doi:10.1111/ppe.13178)
Supplement: Supplementary file 1 — Data S1. [file PPE-39-405-s001.docx]

**Table S1: Components and access instructions for the Euro-Peristat data collection protocol used in the PHIRI project**

| Component | Location and instructions for accessing |
| --- | --- |
| Common Data Model | <https://zenodo.org/records/7639001>  Download and open Zip file “U[seCaseC_v.2.0.0.zip](https://zenodo.org/records/7639001/files/UseCaseC_v.2.0.0.zip?download=1) » which includes  - A spreadsheet in EXCEL format with the data model specification for the use case: 20220304_use_case_C_common_data_model_description_v.2.0.0.xlsx  - An HTML document with a description of Use Case C including the metadata of the data model  - A JSON file in schema.org format including the metadata on the data model in a machine-readable format  - A CSV file with a synthetic data set produced following the specifications of the use case data model for testing purposes |
| R scripts for running the models | <https://zenodo.org/records/10013399>  - LastVersion_core.R : R-script used to collect annual indicators  - LastVersion_permonth.R : R-script used to collect monthly indicators |
| Protocol for data collection | p[rotocol EuroPeristat PHIRI-JAN2023.pdf](https://zenodo.org/records/7639001/files/protocol%20EuroPeristat%20PHIRI-JAN2023.pdf?download=1)  can be downloaded from  <https://zenodo.org/records/7639001> |
| Technical description of process and CDM/Scripts | Zeitlin J, Philibert M, Estupiñán-Romero F, Loghi M, Sakkeus L, Draušnik Ž, Alcaide AR, Durox M, Cap J, Dimnjakovic J, Misins J, Delgado EB, Thissen M, Gissler M, Euro-Peristat Research Group. Developing and testing a protocol using a common data model for federated collection and analysis of national perinatal health indicators in Europe (preprint). Open Research Europe. 2023;https://open-research-europe.ec.europa.eu/articles/3-54. |
| Data tables and report from data collection | <https://www.europeristat.com/publications/european-perinatal-health-report-2015-2019/> |

**Table S2: Data sources used for the common data model and data providers in the Euro-Peristat Network**

|  | **Data sources** | **Data providers** |
| --- | --- | --- |
| Austria | * Birth statistics (Statistics Austria)  * Cause of death statistics (Statistics Austria) | * Statistics Austria |
| Belgium | * Vital Statistics, Statistics Belgium (Statbel) | * Statbel |
| Croatia | * Croatian Medical Birth Database (Croatian Public Health Institute),  * Croatian Mortality Database (Croatian Central Bureau of Statistics) - | * Croatian Institute of Public Health |
| Cyprus | * Medical Birth register (Health Monitoring Unit, Cyprus Ministry of Health)  * Causes of Death register (Health Monitoring Unit, Cyprus Ministry of Health)  * Database for COVID-19 confirmed cases and deaths (Health Monitoring Unit, Cyprus  Ministry of Health) | * Health Monitoring Unit, Ministry of Health |
| Czech Republic | * Institute of Health Statistics and Information of the Czech Republic (national birth register  (mothers and newborns) collecting individual perinatal data.) | * Institute of Health Information and Statistics of the  Czech Republic |
| Denmark | * Medical birth register (The Danish Data authority, Danish Ministry of Health)  * National patient register (The Danish Data authority, Danish Ministry of Health)  * Danish causes of death register (The Danish Data authority, Danish Ministry of Health)  * The Centralized Civil Register | * Statistics Denmark |
| Estonia | * Estonian Medical Birth Register (National Institute for Public Health) was linked with data from  * Estonian Cause of Death Register (National Institute for Public Health) | * Estonian Institute for Population Studies, Tallinn  University |
| Finland | * Medical Birth Register (Finnish Institute for Health Welfare) linked with Central Population  Register (Digital and Population Data Services Agency) and Cause of Death Register  (Statistics Finland)  * Register on Induced Abortions (Finnish Institute for Health Welfare) for late terminations  22–24 weeks | * Finnish Institute for Health and Welfare (THL) |
| France | * Hospital discharge data ( *Programme de Médicalisation des Systèmes d'Information* (PMSI))  in the French National System of Health Data ( *Système national des données de santé (*SDNS)) | * Department for Research, Studies, Assessment  and Statistics (DREES), French Ministry of Health |
| Germany | * IQTIG (Federal Institute for the Quality of Medical Care)  * Destatis (Federal Statistical Office) | * IQTIG |
| Iceland | * The Icelandic Birth Registration  * Hospital register (National University Hospital) | * National University Hospital |
| Ireland | *National Perinatal Reporting System (the Healthcare Pricing Office) | * Healthcare Pricing Office |
| Italy | * Birth certificates (Ministry of Health)  * Causes of deaths (Istat)  * Terminations of pregnancies (Istat)  * Miscarriages (Istat) | * Italian National Institute for Statistics-ISTAT |
| Latvia | * Newborn Register of Latvia (Centre for Disease Prevention and Control of Latvia)  * Register of Causes of Death (Centre for Disease Prevention and Control of Latvia) | * The Centre for Disease Prevention and Control of  Latvia |
| Lithuania | * Medical Date of Births (Institute of Hygiene Health Information Centre)  * Database of the Demographic Statistics (Central Statistical Office)  * Causes of Death register (Institute of Hygiene Health Information Centre) | * Institute of Hygiene, Health Information Centre |
| Luxembourg | * Perinatal Health Monitoring System (Luxembourg Institute of Health)  * National Causes of Death Registry (Directorate of Health of Luxembourg) | * Department of Population Health, Luxembourg  Institute of Health  * Directorate of Health of Luxembourg |
| Malta | * National Obstetrics Information System (Directorate for Health Information and Research)  * National Mortality Register (Directorate for Health Information and Research) | * Directorate for Health Information and Research |
| Netherlands | * Perined (The Netherlands Perinatal Registry) | * Perined |
| Norway | * Medical Birth Register of Norway (The Norwegian Institute of Public Health) | * The Norwegian Institute of Public Health |
| Poland | * Central Statistical Office  * Ministry of Health | * National Research Institute of Mother and Child |
| Portugal | * Instituto Nacional de Estatística – Portugal (Statistics Portugal)  * Central Administration of the Health System | * Institute of Public Health of the University of Porto |
| Romania | * National Institute for Public Health Romania | * National Institute of Public Health Romania |
| Slovakia | *National Health Information Center | * National Health Information Center |
| Slovenia | *Perinatal information system (National institute of public health) | * University Medical Centre, Research Unit |
| Spain | * Vital Statistics (National Statistics Office)  * Specialized Care Registry - Minimum Basic Data Set (Ministry of Health) | * Senior Statistical State Corps and Public Health and  Addictions Directorate, Generalitat Valenciana |
| Sweden | * Medical Birth Register (The National Board of Health and Welfare) | * The National Board of Health and Welfare |
| Switzerland | * Vital Statistics (BEVNAT) | * Swiss Federal Statistical Office |
| UK, all | * MBRRACE UK (University of Oxford and University of Leicester) | * University of Leicester, MBRRACE-UK collaboration |
| UK, England, and Wales | *UK, Office for National Statistics (Live birth and stillbirth registration in England and Wales,  birth notification in England and Wales) | * Office for National Statistics |
| UK, Northern Ireland | * Northern Ireland Maternity System - NIMATS | *Public Health Agency (Northern Ireland) |
| UK, Scotland | * Scottish Morbidity Record 02 (maternity hospital discharge record)  * National Records of Scotland Stillbirth, live birth, and infant death registrations (statutory  vital event registration) | * Public Health Scotland |
| UK, Wales | *Digital Health and Care *Wales* | * Digital Health and Care *Wales* ( *DHCW*) |

**Supplement table S3: Availability of indicators for countries who implemented the PHIRI protocol**

| COUNTRY | Perinatal  variables | Neonatal/  infant death | Mode of delivery | SES | Total number | Number missing |
| --- | --- | --- | --- | --- | --- | --- |
| **Austria** | 8 | 2 | 5 | 1 | 16 | 0 |
| **Belgium** | 8 | 2 | 5 | 1 | 16 | 0 |
| **Croatia** | 8 | 2 | 5 | 1 | 16 | 0 |
| **Cyprus** | 8 | 2 | 5 | 1 | 16 | 0 |
| **Czech Republic** | 8 | 2 | 5 | 1 | 16 | 0 |
| **Denmark** | 8 | 2 | 5 | 1 | 16 | 0 |
| **Estonia** | 8 | 2 | 5 | 1 | 16 | 0 |
| **Finland** | 8 | 2 | 5 | 0 | 15 | 1 |
| **France** | 7 | 0 | 4 | 1 | 12 | 4 |
| **Germany** | 8 | 0 | 5 | 0 | 13 | 3 |
| **Iceland** | 8 | 1 | 5 | 0 | 14 | 2 |
| **Ireland** | 7 | 1 | 2 | 1 | 11 | 3 |
| **Italy** | 8 | 0 | 5 | 1 | 14 | 2 |
| **Latvia** | 8 | 1 | 5 | 1 | 15 | 1 |
| **Lithuania** | 8 | 1 | 5 | 1 | 15 | 1 |
| **Luxembourg** | 8 | 0 | 5 | 1 | 14 | 2 |
| **Malta** | 8 | 1 | 5 | 1 | 15 | 1 |
| **Norway** | 8 | 2 | 5 | 0 | 15 | 1 |
| **Poland** | 8 | 2 | 0 | 1 | 11 | 5 |
| **Portugal** | 8 | 0 | 0 | 1 | 9 | 7 |
| **Romania** | 7 | 2 | 0 | 1 | 10 | 6 |
| **Slovakia** | 8 | 0 | 5 | 1 | 14 | 2 |
| **Slovenia** | 8 | 1 | 5 | 1 | 15 | 1 |
| **Spain** | 8 | 0 | 1 | 1 | 10 | 6 |
| **Sweden** | 8 | 2 | 5 | 0 | 15 | 1 |
| **Switzerland** | 8 | 2 | 0 | 1 | 11 | 5 |
| **The Netherlands** | 8 | 1 | 5 | 1 | 15 | 1 |
| **UK** | 8 | 1 | 0 | 1 | 10 | 6 |
| England and Wales | 7 | 2 | 0 | 0 | 9 | 7 |
| Northern Ireland | 8 | 1 | 5 | 1 | 15 | 1 |
| Scotland | 8 | 2 | 5 | 1 | 16 | 0 |

NOTE: Perinatal variables (gestational age, birthweight, sex, type of pregnancy, vital status at birth, maternal age, parity, previous caesarean delivery), Infant/neonatal death (early neonatal, late neonatal, infant mortality), Mode of delivery ((CS vs vaginal), type of caesarean, instrumental delivery, onset of labour, presentation of the baby), SES.

**Supplement table S4: Data validation and correction**

| **Country** | **What procedures are used to check data quality** | **Can the register keeper contact data providers for further information and corrections** | **Please describe:** | **Are the old years updated in cases of new data, updated data or observed mistakes** | **Comment** | **How long the database is open for new entries (i.e. is the year ˝locked˝ at some point)** |
| --- | --- | --- | --- | --- | --- | --- |
| Austria | plausibility checks  checks on missing data | Yes | Missing information on birthweight, gestational age and delivery mode are always requested | Yes | yes, in case mistakes are observed | <1 year |
| Belgium | Checks were carried out at the regional level (through contacts with maternity hospitals) and then at the Statbel level, with consistency checks. | Yes | Statbel can contact the regional bodies responsible for data collection, which in turn can review the records or contact the maternity hospitals. | No |  | 1 to 2 years |
| Croatia | First level of control are county public health institutes, which control the data of institutions within their county. The second step of work on quality is Croatian Institute of Public Health, where the quantity and quality of collected data is controlled. | Yes | In case of ambiguities, both levels of verification, if necessary, can contact the hospital from which the data was collected. | Yes |  | No locked time |
| Cyprus | Quality checks through formulas and validation rules in Excel and Access | Yes | When the information is entered electronically, it is checked and for corrections there is a continuous communication with the data providers | Yes |  | No locked time |
| Czech Republic | regular monthly reports  linkage of reports on mother and child  comparisons with health insurance data | Yes | register has a legal right to check data providers (registers are obligatory) | No |  | <1 year |
| Denmark | Internal consistency of recordings | No |  | Yes |  | No locked time |
| Estonia | Registry makes initial controls and in case of inconsistencies turns to data providers who can go back to original documents | Yes | Registry turn to data provider who can check original documents based on which the information has been entered into the registry card. | Yes | In case it occurs during the period until disclosure, it is updated, Otherwise it is updated and when one gets the data anew, it is with corrections. | No locked time |
| France | plausibility checks  Procedures checking consistency of data between mother and baby | yes | if necessary, can contact the hospital from which the data was collected. | yes | yes, in case mistakes are observed | No locked time |
| Finland | All key variables are checked and missing or incorrect data are double-checked from the birth hospital.  Further, some variables can be complete from other registers, e.g. number of prenatal visits and timing of first prenatal visit. | Yes | There is a contact person in each birth hospital who will be contacted in case of data problems. | Yes |  | No locked time |
| Germany | 1) plausibility checks (internal validation) every year 2) comparison of results in database with clinical charts in a two stage random sample of hospitals and births (approx once in 10 years | Yes, via federal quality agencies | Hospitals whose data show serious fails in plausibility checks are contacted | No | Only if new data (births) have not been collected before, data from these births are stored in the database of the new year | 1 to 2 years |
| Iceland | Unfortunately, these have not been very clearly defined yet. There are mostly two manual check points:  1) Before discharge the summary information and birth certificate should be checked by midwifes, doctors and medical secretaries ascertaining that data is correctly registered.  2) When summary statistics are made, data entries are cross-checked and we look for inconsistencies. Sometimes this leads to validating data entries in the medical records and corrections can be made.  No validation studies are formally published as such, although this has certainly been done in studies involving only births in Landspitali. | Yes | Iceland is a small country so the same doctors working for the Birth Registry also work at the Hospitals that cover most deliveries in Iceland. Otherwise, we can contact other data providers for corrections. | Yes |  | No locked time |
| Ireland | Data quality activities are performed at a hospital level and a national level. Data quality and validation checks are run monthly and annually on hospital level data. Separate annual data quality checks are performed on national NPRS data. Quality checks include the application of a comprehensive series of edits, which are built into the custom designed NPRS data entry software to check for completeness, validity, logic, and coding errors. Any feedback or queries arising from the validation checks are sent back to hospital/midwife for review. The HPO is introducing an additional validation check in conjunction with the Newborn Hearing Screening Programme to check the home births on a monthly basis. | Yes | incorrect or missing information is sent back to the data providers for clarification | Yes |  | 1 to 2 years |
| Italy | Procedures checking consistency of data by crossing variables and comparing with other data sources | Yes | Contact with data providers is guaranteed during phases of check data quality | Yes |  | 1 to 2 years |
| Latvia | 1) The program has built-in logical controls that prevent some input errors, as well as mandatory fields;  2) Regularly comparison of total newborn and delivery data with institutions;  3) Comparison of stillbirths and early neonatal deaths with the causes of death database;  4) if data quality errors are noticed (e.g. inappropriately low weight at the specified gestational age, etc.), it is clarified from the data provider whether the data is correct etc. | Yes | CDPC has contracts with institutions for data entry, there is close cooperation with the direct data providers of maternity units, therefore unclear questions or errors are corrected during communication, as well as regular comparison of the number of births in institutions. | Yes |  | <1 year |
| Lithuania | Paper forms of Newborns or Stillbirths we receive from obstetrics departments   Information is putting into our original software. There are a lot of checking: Robson group (include mode of delivery, gestational age, parity, previous CS, type of CS, type of pregnancy, onset), mortality in first month, mother‘s age, Rh(D) negative mothers and Anti-D immuno prophylaxis, birthweight, head circumference, ICD-codes and others condition:   Every record is checking. If any information is not correct – will create a error‘s list. Then we connect with specialists of obstetrics departments for correction | Yes |  | Yes | it is possible to update data of the old years | No locked time |
| Luxembourg | Exhaustiveness verification by comparison with hospital registries, coherence control of data (during typing by input rules and a posteriori by dedicated checks), missing information verification with hospitals. Data is sent by hospitals, then cleaned and quality-tested for the first time, then sent back to hospital for completion/correction and verification. Finally, the data are sent back to  Luxembourg Institute of Health and quality-tested for the second time before final validation. | Yes | Data are provided with pseudonyms, which are removed after validation. | No |  | 1 to 2 years |
| Malta | A comprehensive list of data validations is run annually. | Yes | We can contact the dedicated staff collecting data at the hospitals and ask for checks and clarifications where needed | Yes | Only updated until 9 months post end of year | <1 year |
| Netherlands | Every month a report with missing or unexpected values in the data is send the care providers. Additionally, the software used by care providers performs automatic checks. Finally, trends are reviewed on a quarterly basis. | Yes |  | Yes |  | No locked time |
| Northern Ireland | Inbuilt data validation checks  Following input, data quality reports run regularly | Yes | Yes, it is possible if an invalid entry is noticed that it can be checked and resolved with data owner | Yes |  | No locked time |
| Norway | All reported live births are double-checked with the National Population Register and missing births are required from the delivery units. Number of reported stillbirths and spontaneous abortions are checked routinely against numbers at each delivery unit, and missing abortions/stillbirths asked for. All deliveries outside institutions are double checked. All key variables in the registry are checked for inconsistency, missing or incorrect information based on several data “quality rules”. Inconsistent, missing, and erroneous data are double-checked from the delivery unit. | Yes | The delivery units are contacted for all notified births where the information is not according to the registry’s defined “quality rules”, as described above. | Yes |  | No locked time |
| Poland | This is on the side of Central Statistical Office and the civil registration system of births and deaths.  For example, there is a system of verification of causes of deaths.  I work on the data provided to guarantee the data comparability. | Yes | There is a system of verification of causes of deaths | No | Rather not. | 1 to 2 years |
| Portugal | Evaluation of death certificates  Births certificates accepted as they are | Yes | In case of errors, they can come back to ask for correction (obvious errors)  From time to time, they evaluate a sample, with a more in-depth evaluation | Yes |  | There is a locked time |
| Scotland | On a monthly basis, the Maternity team produce data quality tables to monitor SMR02 completeness and frequency of use of the not known option for the most commonly used data variables. This and any other data issues are monitored by the PHS Data Management team who may then raise with colleagues in NHS Boards. | Yes | Data Management provides a data support and monitoring service to NHS Boards and other health care data providers in the submission of national data sets including SMR02. Their key responsibilities include: monitoring data submissions in terms of completeness and timeliness; liaising with data providers to ensure data is validated against national standards and where appropriate highlight any issues and ensure they are resolved; investigating any data quality issues highlighted and work with data providers and system suppliers as required to resolve them; ensuring that all amendments required to resolve data quality issues are applied either by the data providers themselves or by PHS on their behalf; liaising regularly with data providers to ensure issues affecting or likely to affect data submission are identified at an early stage and addressed. | Yes | SMR02s may contain a mix of records that are “inserts” (never submitted before), “replacements” (submitted previously and have been updated) and occasionally “deletes” (records to be removed). As far as 'inserts' are concerned there is no cut-off for submitting late data, SMR files remain open. The same used to be true for 'replacements' however a restriction was put in place from April 2020 so that Boards can only re-submit records within 2 years of the discharge date. Data Management can upload replacement records on behalf of Boards if amendments are required after the 2 years window has passed. | No locked time |
| Slovenia | No fixed procedures. Few years ago, there was study dedicated to data quality. | Yes | in case of disagreement between perinatal system and death certificates | No |  | <1 year |
| Spain | Not known. Procedures are applicated by INE | No |  | No |  | 1 to 2 years |
| Sweden | Numerous procedures, including cross-checks with Statistics Sweden to minimize lost-to-follow-up | Yes |  | Yes |  | No locked time |
| Switzerland | civil registry checks as well as plausibility checks for birthweight and gestational age | Yes |  | No |  | <1 year |
| UK- England & Wales | Plausible birth weights by gestational age and sex. Missing data are no longer imputed (as of 2018) due to very small number of missing values. | Yes | Various validation checks by registrar during face-to-face registration process | Yes | If errors are identified which are sufficient to change the interpretation | 14 months |
| UK-MBRRACE | Validation checks for data entry, linkage to birth and death notifications for ensuring data ascertainment and validation of data items | Yes | In constant contact to ensure completeness of data | Yes | Data are updated but report data for individual years held static | No locked time |

**Supplement Table S5: Annual report or website on published data**

| **Country** | **If there is an annual report or website where the data are published, please provide link(s):** |
| --- | --- |
| Austria | <https://www.statistik.at/en/statistics/population-and-society/population/births/medical-and-socio-medical-characteristics-of-newborns> |
| Belgium | There is an annual publication on the website of Statbel (Very general publication) : <https://statbel.fgov.be/fr>  More detailed exploitation of the basic data is carried out by the regional institutions whose remit includes health. |
| Croatia | Croatian Health Statistics Yearbook: <https://www.hzjz.hr/wp-content/uploads/2023/05/HZSLj_-_2021_v._05.2023..pdf> Childbirths in healthcare institutions (report): <https://www.hzjz.hr/wp-content/uploads/2022/07/Porodi_2021_12082022.pdf> Infants deaths in Croatia (report): <https://www.hzjz.hr/wp-content/uploads/2022/12/Dojenacke_smrti_-_2021pdf> |
| Cyprus | <https://www.moh.gov.cy/moh/moh.nsf/All/1A26898D0CD56210C22579C600278E4B?OpenDocument>  <https://www.moh.gov.cy/moh/moh.nsf/All/72DC28C8685DE0AAC22579C60026667B?OpenDocument> |
| Czech Republic | [www.uzis.cz](http://www.uzis.cz) |
| Denmark | https://www.dst.dk/en/Statistik/emner/borgere/befolkning/foedsler |
| Estonia | Until 2016 there has been a bi-annual report on Birth and Abortion Registries. Eesti Meditsiiniline Sünniregister 1992–2016. Eesti Abordiregister 1996–2016 <https://www.tai.ee/sites/default/files/2021-03/152119585973_S%C3%BCnniregister_Abordiregister_2016.pdf>  On the 30th anniversary of the birth registry a special number of journal Eesti Arst was issued (Eesti Arst 2022;101(Lisa 6):1–44) <https://www.tai.ee/sites/default/files/2022-11/Meditsiiniline_synniregister_EestiArst_erinumber_2022.pdf>  Since 2017 the data is on the website in tables <https://statistika.tai.ee/index_en.html> |
| Finland | <https://thl.fi/en/web/thlfi-en/statistics-and-data/statistics-by-topic/sexual-and-reproductive-health/parturients-deliveries-and-births/perinatal-statistics-parturients-delivers-and-newborns> |
| France | Scan santé (The SNDS data used to construct the database are provided here)  Data.drees ([Indicateurs de santé périnatale — DATA.DREES (solidarites-sante.gouv.fr)](https://data.drees.solidarites-sante.gouv.fr/explore/dataset/1520_indicateurs-de-sante-perinatale/information/) |
| Germany | <https://iqtig.org/qs-verfahren/qs-pm/> |
| Iceland | Overview statistics: <https://island.is/faedingar-tolur>  Annual reports: <https://island.is/faedingar-tolur/arsskyrslur-faedingaskraningar>  Team specific statistics reports from the Birth registry: <https://island.is/faedingar-tolur/talnabrunnur-faedingar> |
| Ireland | <https://www.hpo.ie/> |
| Italy | For miscarriages and neonatal/infant mortality : Istat Datawarehouse portal :<http://dati.istat.it/>  For TOPs : Istat Datawarehouse portal :<http://dati.istat.it/> ; Annual Report to Parliament by Ministry of Health: <https://www.salute.gov.it/portale/donna/dettaglioPubblicazioniDonna.jsp?lingua=italiano&id=3367>  For births: Annual Report on Deliveries by Ministry of Health: https://www.salute.gov.it/portale/documentazione/p6_2_2_1.jsp?lingua=italiano&id=3346 |
| Latvia | There are no specific report but all data are available in Health Statistics database: <https://statistika.spkc.gov.lv/pxweb/en/Health/> |
| Lithuania | <https://www.hi.lt/sveikatos-statistikos-leidiniai/#--gimimu-medicininiai-duomenys> |
| Luxembourg | Triennial reports, available at <https://susana.lu/web/Publications/Rapports.aspx> |
| Malta | <https://healthservices.gov.mt/en/dhir/Pages/Registries/births.aspx> |
| Netherlands | [www.peristat.nl](http://www.peristat.nl) |
| Northern Ireland | https://www.publichealth.hscni.net/directorates/operations/statistics |
| Norway | Annual report from the Abortion registry: <https://www.fhi.no/publ/2023/rapport-om-svangerskapsavbrot--2022/>  The website for the Statistics bank at the MBRN: <https://statistikkbank.fhi.no/mfr/> |
| Poland | These data are published yearly in the Demographic Yearbooks, according to the year of registration (not birth) : <https://stat.gov.pl/obszary-tematyczne/roczniki-statystyczne/roczniki-statystyczne/rocznik-demograficzny-2021,3,15.html> |
| Portugal | [Portal do INE](https://www.ine.pt/xportal/xmain?xpid=INE&xpgid=ine_main)  [PORDATA - Estatísticas, gráficos e indicadores](https://www.pordata.pt/) |
| Scotland | <https://www.publichealthscotland.scot/publications/births-in-scotland> |
| Slovenia | on web pages of institute of public health: <https://nijz.si/wp-content/uploads/2022/10/prva_objava_pis_2022_0.pdf> |
| Spain | <https://www.ine.es/dyngs/INEbase/es/operacion.htm?c=Estadistica_C&cid=1254736177007&menu=resultados&idp=1254735573002> |
| Sweden | <https://www.socialstyrelsen.se/statistik-och-data/statistik/alla-statistikamnen/graviditeter-forlossningar-och-nyfodda/> |
| Switzerland | <https://www.bfs.admin.ch/bfs/fr/home/statistiques/sante/etat-sante/sante-nouveau-nes.html> <https://www.bfs.admin.ch/bfs/fr/home/statistiques/population/naissances-deces/naissances.html> |
| UK-MBRRACE | <https://www.npeu.ox.ac.uk/mbrrace-uk/reports/perinatal-mortality-surveillance> |

**Supplement Table S6: Example of recent publications using the data**

| **Country** | **Please give an example of recent publication using the data:** |
| --- | --- |
| Austria | Different research institutions use the data, i.e. Department of Epidemiology at the Centre of Public Health,. Medical University of Vienna. |
| Belgium | Research is done by the researchers from School of Public Health  and Faculty of medecine, Université libre de Bruxelles (ULB). |
| Croatia | Rodin U, Barišić I, Cerovečki I, Draušnik Ž. Utjecaj COVID-19 na perinatalno zdravlje, zdravlje majki i dostupnost perinatalne zdravstvene zaštite u Hrvatskoj. Zbornik radova XXXIV. Perinatalni dani „Ante Dražančić“. Zagreb: HLZ HDPM; 2023. |
| Cyprus | Causes of Death in Cyprus for year 2022: <https://www.moh.gov.cy/moh/moh.nsf/All/72DC28C8685DE0AAC22579C60026667B?OpenDocument> |
| Czech Republic | European Perinatal Health Report;  Neonatal mortality risk for vulnerable newborn types in 15 countries using 125.5 million nationwide birth outcome records, 2000 to 2020 Journal: BJOG: An International Journal of Obstetrics and Gynaecology |
| Denmark | <https://www.sciencedirect.com/science/article/pii/S000293782101231X> |
| Estonia | Our linked data is used for EURO_PERISTAT network publications and these can be referred to from eurperistat.com/publications list. Separately research is done on Medical Birth Registry e.g. Veber, Triin; Dahal, Usha; Lang, Katrin; Orru, Kati; Orru, Hans (2022). Industrial Air Pollution Leads to Adverse Birth Outcomes: A Systematized Review of Different Exposure Metrics and Health Effects in Newborns. Public Health Reviews, 43, 1604775. DOI: 10.3389/phrs.2022.1604775. Luhamaa, Katre; McEwan-Strand, Amy; Ruiken, Barbara; Skivenes, Marit; Wingens, Florian (2021). Services and support to mothers and newborn babies in vulnerable situations. A study of eight countries. Children and Youth Services Review, 120, 105762. DOI: 10.1016/j.childyouth.2020.105762. Franková V, Driscoll RO, Jansen ME, Loeber JG, Kožich V, Bonham J, Borde P, Brincat I, Cheillan D, Dekkers E, Fingerhut R, Kuš IB, Girginoudis P, Groselj U, Hougaard D, Knapková M, la Marca G, Malniece I, Nanu MI, Nennstiel U, Olkhovych N, Oltarzewski M, Pettersen RD, Racz G, Reinson K, Salimbayeva D, Songailiene J, Vilarinho L, Vogazianos M, Zetterström RH, Zeyda M; Members of the European Society of Human Genetics (ESHG)-EuroGentest Quality Sub-Committee. (2020). Regulatory landscape of providing information on newborn screening to parents across Europe. European Journal of Human Genetics. |
| France | Fresson J, Bruckner TA, Ray CL, Goffinet F, Rey S, Blondel B, Deneux-Tharaux C, Ancel PY, Zeitlin J. Decreases in preterm birth during the first COVID-19 lockdown in France by gestational age sub-groups and regional COVID-19 incidence. Ann Epidemiol. 2022;72:74-81. |
| Finland | Vilkko R, Räisänen S, Gissler M, Stefanovic V, Kalliala I, Heinonen S: Busy day effect on adverse obstetric outcomes using a nationwide ecosystem approach: cross-sectional register study of 601 247 hospital deliveries. BJOG: An International Journal of Obstetrics and Gynaecology 2023. doi: 10.1111/1471-0528.17502 |
| Germany | Heller G, Bauer E, Schill S, Thomas T, Louwen F, Wolff F, Misselwitz B, Schmidt S, Veit C : Decision-to-Delivery Time and Perinatal Complications in Emergency Cesarean Section. Dtsch Arztebl Int 2017; 114: 589-96. DOI: 10.3238/arztebl.2017.0589  <https://www.aerzteblatt.de/int/archive/article/193157> |
| Iceland | Swift EM, Gunnarsdottir J, Zoega H, Bjarnadottir RI, Steingrimsdottir T, Einarsdottir K. Trends in labor induction indications: A 20-year population-based study. Acta Obstet Gynecol Scand. 2022 Dec;101(12):1422-1430. doi: 10.1111/aogs.14447. |
| Ireland | <https://www.esri.ie/publications/unequal-chances-inequalities-in-mortality-in-ireland> |
| Italy | National Observatory on Health in Italian Regions (Osservasalute) 2022: Salute riproduttiva: un confronto tra i Paesi dell’Unione Europea [Reproductive health: a comparison among European Union countries] pag.543. [https://osservatoriosullasalute.it/osservasalute/rapporto-osservasalute-2022](https://urldefense.com/v3/__https:/osservatoriosullasalute.it/osservasalute/rapporto-osservasalute-2022__;!!OmMtAkQYH20LOJIZAzSBroM!-SItal9jbpsaXebrS3ybnJWTGoo7bA5eJKmklM3UC9ZqQvyKmV0BAzFUSsOl1VzGpUjRa8CckRH2yWA$) |
| Latvia | The data is used by different research institutions and universities. An example:  <https://www.ncbi.nlm.nih.gov/pmc/articles/PMC11123435/>  <https://pubmed.ncbi.nlm.nih.gov/31266254/>  <https://pubmed.ncbi.nlm.nih.gov/30112211/> |
| Lithuania | REGIONAL REFERENCES VS. INTERNATIONAL STANDARDS FOR ASSESSING WEIGHT AND LENGTH BY GESTATIONAL AGE IN LITHUANIAN NEONATES, by Ruta Morkuniene, Tim Cole, Egle Marija Jakimaviciene, Agne Bankauskiene, Jelena Isakova, Nijole Drazdiene, Vytautas Basys, Janina Tutkuviene, published in Frontiers in Pediatrics, section Neonatology. To view the online publication, please click here: http://journal.frontiersin.org/article/10.3389/fped.2023.1173685/full?&utm_source=Email_to_authors_&utm_medium=Email&utm_content=T1_11.5e1_author&utm_campaign=Email_publication&field=&journalName=Frontiers_in_Pediatrics&id=1173685 |
| Luxembourg | Euro-Peristat publications, EuroNeoNet, scientific conferences, surveillance reports |
| Malta | Hili, C., Savona-Ventura, C. and Xuereb, R.B., 2023. The Perinatal Outcomes of Maltese and Sub-Saharan African Migrant Women: A Comparative, Retrospective Study. International Journal of Childbirth. |
| Netherlands | The data is used by different research institutions and universities.  An example:  Journal of Perinatology; https://doi.org/10.1038/s41372-023-01786-2 |
| UK: Northern Ireland | Annual maternity reports - [Statistics \| HSC Public Health Agency (hscni.net)](https://www.publichealth.hscni.net/directorates/operations/statistics)  Data used regularly by health professionals, researchers etc. |
| Norway | The MBRN data are used in very many scientific publications each year. An overview of publications can be found here: [*https://www.fhi.no/op/mfr/publikasjonsliste-for-medisinsk-fodselsregister/*](https://www.fhi.no/op/mfr/publikasjonsliste-for-medisinsk-fodselsregister/)  Some examples using data from the MBRN linked to other data sources:   1. Paramsothy A, Hegvik TA, Engeland A, Bjørge T, Egeland GM, Klungsøyr K.  [*Fetal Exposure to Preeclampsia and Later Risk of Cardiometabolic Disorders: A Population-Based Cohort Study.*](https://pubmed.ncbi.nlm.nih.gov/37737002/) *Hypertension. 2023 Nov;80(11):e158-e166. doi: 10.1161/ HYPERTENSIONAHA. 122.20682.* (Linked data between MBRN and the Norwegian Prescription Database (NorPD)) 2. Sima YT, Skjaerven R, Kvalvik LG, Morken NH, Klungsøyr K, Mannseth J, Sørbye LM. ***Birth Weight in Consecutive Pregnancies and Maternal Cardiovascular Disease Mortality Among Spontaneous and Iatrogenic Term Births: A Population-Based Cohort Study.*** *Am J Epidemiol. 2023 Aug 4;192(8):1326-1334. doi: 10.1093/aje/kwad075.* (Linked data between MBRN, the Cause of Death Registry and the Education Database) 3. Hegvik TA, Klungsøyr K, Kuja-Halkola R, Remes H, et al. [***Labor epidural analgesia and subsequent risk of offspring autism spectrum disorder and attention-deficit/hyperactivity disorder: a cross-national cohort study of 4.5 million individuals and their siblings****.*](https://pubmed.ncbi.nlm.nih.gov/35973476/) *Am J Obstet Gynecol. 2022 Aug 13:S0002-9378(22)00650-0. doi: 10.1016/j.ajog.2022.08.016.* (Linked data (on births, diagnoses and prescriptions) from Finland and Norway) 4. Solberg BS, Hegvik TA, Halmøy A, Skjaerven R, Engeland A, Haavik J, Klungsøyr K. [***Sex differences in parent-offspring recurrence of attention-deficit/hyperactivity disorder.***](https://pubmed.ncbi.nlm.nih.gov/33341963/) *J Child Psychol Psychiatry. 2021 Aug;62(8):1010-1018. doi: 10.1111/jcpp.13368. PMID: 33341963*(Linked data between MBRN, Norwegian Patient Registry and the NorPD) 5. Daltveit DS, Klungsøyr K, Engeland A, ….., Bjørge T. [***Cancer risk in individuals with major birth defects: large Nordic population based case-control study among children, adolescents, and adults.***](https://pubmed.ncbi.nlm.nih.gov/33268348/) *BMJ. 2020 Dec 2;371:m4060. doi: 10.1136/bmj.m4060.* (Nordic study using linked data from birth registries and cancer registries) 6. Kvalvik LG, Wilcox AJ, Skjærven R, Østbye T, Harmon QE. [Term complications and subsequent risk of preterm birth: registry based study.](https://pubmed.ncbi.nlm.nih.gov/32349968/) *BMJ. 2020 Apr 29;369:m1007. doi: 10.1136/bmj.m1007. PMID: 32349968*(Linked data between MBRN and the Education Database) |
| Poland | These data are used mainly by demographers. An example: Kotowska, I. E. (2020). Uwagi o urodzeniach i niskiej dzietności w Polsce oraz polityce rodzinnej wspierającej prokreację. Studia Demograficzne, (2(176), 11-29. <https://doi.org/10.33119/SD.2019.2.1> |
| Portugal | No response. |
| Scotland | Routine annual reports: [Maternity and births - Early years and young people - Our areas of work - Public Health Scotland](https://www.publichealthscotland.scot/our-areas-of-work/early-years-and-young-people/maternity-and-births/)  An example of how data were used: [SARS-CoV-2 infection and COVID-19 vaccination rates in pregnant women in Scotland \| Nature Medicine](https://www.nature.com/articles/s41591-021-01666-2)  The data are used frequently by researchers, universities and in projects that are outwith the scope of analysts in the Maternity team. In these circumstances we would provide the data extracts but not necessarily be aware of published papers. |
| Slovenia | Lucovnik M, Verdenik I, Stopar Pintaric T. Intrapartum Cesarean Section and Perinatal Outcomes after Epidural Analgesia or Remifentanil-PCA in Breech and Twin Deliveries. Medicina (Kaunas). 2023 May 25;59(6):1026. doi: 10.3390/medicina59061026.  Pečlin P, Kovač L, Tul N, Verdenik I, Bregar AT. Comparison of "growth promoted" and "normally grown" dichorionic-diamniotic twins: A population-based study. Eur J Obstet Gynecol Reprod Biol X. 2022 May 23;15:100154. doi: 10.1016/j.eurox.2022.100154.  Esih K, Trunk T, Osredkar D, Verdenik I, Neubauer D, Troha Gergeli A, Lučovnik M. The impact of birthweight on the development of cerebral palsy: A population-based matched case-control study. Early Hum Dev. 2022 Feb;165:105533. doi: 10.1016/j.earlhumdev.2021.105533.  and many more |
| Spain | Recio Alcaide, A., Perez Lopez, C., & Bolúmar, F. (2022). Influence of sociodemographic factors in birth seasonality in Spain. American Journal of Human Biology, 34(10), e23788. |
| Sweden | Register used for a large number of scientific publications every year as illustrated below by several most recent recent publications:   - Bergman K, Svanvik T, Basic C, Rosengren A, Zverkova Sandström T, Celind J, Sjöland H, Wikström AK, Schaufelberger M, Thunström E Heart disease in pregnancy and risk of pre-eclampsia: a Swedish register-based study. Open Heart. 2024 May 23;11(1):e002728. - Lundborg L, Ananth CV, Joseph KS, Cnattingius S, Razaz N.Changes in the prevalence of maternal chronic conditions during pregnancy: A nationwide age-period-cohort analysis. BJOG. 2025 Jan;132(1):44-52. - Tsamantioti E, Sandström A, Lindblad Wollmann C, Snowden JM, Razaz N. Association of Severe Maternal Morbidity With Subsequent Birth. JAMA. 2024 Nov 25:e2420957. - Gadsbøll C, Björklund LJ, Norman M, Abrahamsson T, Domellöf M, Elfvin A, Farooqi A, Hellström-Westas L, Håkansson S, Källén K, Normann E, Serenius F, Sävman K, Um-Bergström P, Ådén U, Ley D Centralisation of extremely preterm births and decreased early postnatal mortality in Sweden, 2004-2007 versus 2014-2016. .Acta Paediatr. 2024 Sep 23 |
| Switzerland | Euro-Peristat Research N. Population birth data and pandemic readiness in Europe. *BJOG*. Sep 27 2021;129(2):179-84. doi:10.1111/1471-0528.16946  Calvert C, Brockway MM, Zoega H, et al. Changes in preterm birth and stillbirth during COVID-19 lockdowns in 26 countries. *Nat Hum Behav*. 2023;7(4):529-544. doi:10.1038/s41562-023-01522-y |
| UK-MBRRACE | Matthews RJ, Draper ES, Manktelow BN, Kurinczuk JJ, Fenton AC, Dunkley-Bent J, Gallimore I, Smith LK; MBRRACE-UK Collaboration. Understanding ethnic inequalities in stillbirth rates: a UK population-based cohort study. BMJ Open. 2022 Mar 9;12(2):e057412. doi: 10.1136/bmjopen-2021-057412. PMID: 35264402; PMCID: PMC8968514.  Smith LK, van Blankenstein E, Fox G, Seaton SE, Martínez-Jiménez M, Petrou S, Battersby C; MBRRACE-UK Perinatal Surveillance Group. Effect of national guidance on survival for babies born at 22 weeks' gestation in England and Wales: population based cohort study. BMJ Med. 2023 Nov 7;2(1):e000579. doi: 10.1136/bmjmed-2023-000579. PMID: 38027415; PMCID: PMC10649719.    Evans MJ, Draper ES, Smith LK. Impact of sociodemographic and clinical factors on offer and parental consent to postmortem following stillbirth or neonatal death: a UK population-based cohort study. Arch Dis Child Fetal Neonatal Ed. 2020 Sep;105(5):532-537. doi: 10.1136/archdischild-2019-318226. Epub 2020 Jan 22. PMID: 31969458. |
